# STITCH enabled protein-protein interactions between *Phyllanthus emblica* and peri-implant microbiome

**DOI:** 10.6026/97320630019491

**Published:** 2023-04-30

**Authors:** Karthickraj Selladurai, Subhashree Rohinikumar, Abhinav Rajendra Prabu, Vishnu Priya Veeraraghavan, Thiyaneswaran Nesappan, Rajalakshmanan Eswaramoorthy

**Affiliations:** 1 Department of Implantology, Saveetha Dental College and Hospitals, Saveetha Institute of Medical and Medical and Technical Sciences, Saveetha University, Chennai 600077,India; 2 Department of Biochemistry, Saveetha Dental College and Hospitals, Saveetha Institute of Medical and Medical and Technical Sciences, Saveetha University, Chennai 600077,India; 3 Department of Biomaterials, Centre of Molecular Medicine and Diagnostics (COMManD), Saveetha Dental College and Hospitals, Saveetha Institute of Medical and Medical and Technical Sciences, Saveetha University, Chennai 600077, India

**Keywords:** *Phyllanthus emblica*, peri -implant microbes, peri implantitis.

## Abstract

It is of interest to document the protein-protein interaction between *Phyllanthus emblica* and peri implant pathogens in the context of peri implant illness. The peri implant pathogens includes *Aggregatibacter actinomycetemcomitans* (D7S-1), *Centipeda periodontii*, *Campylobacter gracilis*, *Fusobacterium nucleatum*, *Slackia exigua*, *Prevotella intermedia*, *Tannerella forsythia*, *Staphylococcus aureus*, *Bacteroides fragilis*, and *Bacteroides fragilis*. Hence, a user-defined query was used to conduct analysis on the provided bacterial strains whose molecular data available in the STITCH database. Thus, we used the STITCH tool to examine protein interactions and the VirulentPred tool to assess pathogenicity using the known molecular data on *Phyllanthus emblica* and peri implant pathogens. Data shows that *Phyllanthus emblica* interacts with peri implant pathogens.

## Background:

**Figure 1:**
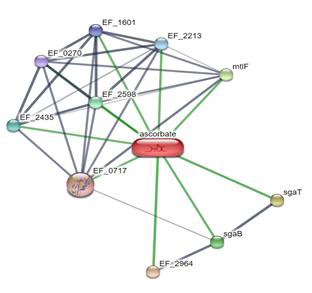
Interactions of ascorbic with various proteins against the various oral microbes

**Figure 2:**
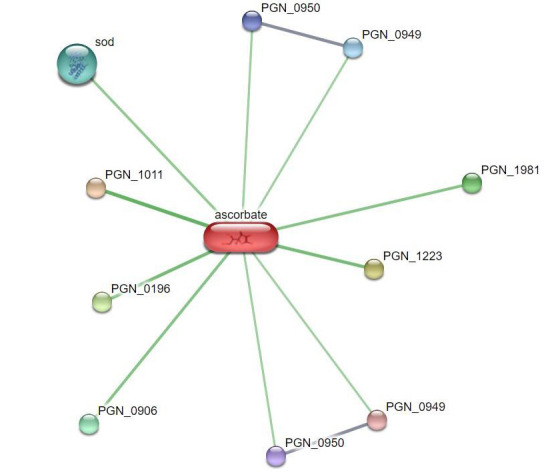
Interactions of ascorbic with various proteins against the various oral microbes

Dental implants are almost completely replacing tooth-supported fixed prostheses. The survival rates of implants range from 96.4% to 99% [1]. The issue is lengthy healing time compared to fixed prostheses. A crucial problem for the implant's longevity is maintenance. The chance of losing additional implants is due to peri-implant disease [2, 3, 4, 5, 6, 7, 8, 9, 10, 11, 12]. Therefore, it is of interest to document the protein-protein interaction between *Phyllanthus emblica* and peri implant pathogens in the context of peri implant illness (Table 1 and Figures 1 and 2).

## Materials and methods:

## Study design:

It is of interest to study the interaction of Phylanthus emblica with *Aggregatibacter actinomycetemcomitans* (D7S-1), *Centipeda periodontii*, *Campylobacter gracilisi*, *Fusobacterium nucleatum*, *Slackia exigua*, *Prevotella intermedia*, Tannerella forsythia, *Staphylococcus aureus*, *Bacteroides fragilis*, and *Bacteroides fragilis* using known molecular data. Hence, a user-defined query was used to conduct analysis on the provided bacterial strains whose molecular data available in the STITCH database [2, 13].

## Prediction of bacterial protein and metal oxide interactions:

The STITCH database (Version 5) (Szklarczyk D *et al*. 2016) [14] is an open-source platform with an extensive collection of data about interactions, both physical and functional associations made possible by computational prediction of interactions from primary databases, the repertoire of proteins which interact with A. *actinomycetemcomitans* (D7S-1), *B. fragilis* (ATCC 25285), *C. gracilis* (RM 3268), *F. nucleatum* (ATCC (ATCC43037).

## Prediction of subcellular localization of the virulent protein:

Cell surface proteins are of particular interest because they can be exploited as novel drug targets. Subcellular localization of proteins aids in the identification of drug targets and could serve as a possible target for new medications. An algorithm called Gneg-mPLoc uses an amino acid sequence to determine the likely location of a protein [14, 15].

## Prediction of subcellular localization of the virulent protein:

Subcellular localization of proteins helps in the identification of drug targets and could serve as a potential target for new medicines [16]. Cell surface proteins are of great interest as they can be used as novel drug targets. Gneg-mPLoc is an algorithm that assigns a probable localization site to a protein from an amino acid sequence provided.

## Results:

In order to classify the results as virulent or avirulent, the STITCH v5 tool was utilized to assess the interaction between the microbe and the element of interest. The protein target derivatives of the reactions were then further analyzed with the VICMpred and Virulentpred algorithms. It has been discovered that the chemical ascorbic interacts with proteins essential for cellular metabolism and other activities. It is intriguing to note that the element of interest also interacted with the peri-implant infections virulence factors. The enzymes glutaminase, glutamine synthetase, and superoxide group of enzymes, which are predominantly involved in nitrogen metabolism and enzymes involved in free radical formation, were implicated in the majority of interactions. The majority of the proteins targeted were discovered to be present at the cytoplasm - periplasm compartment in addition to these predictions, which were based on the subcellular localization of the 10 virulence factors and epitope analysis.

## Discussion:

The microflora on initial examination was thought to be similar to that of periodontitis more specifically the red complex bacteria [17], initial colonisation of peri-implant surfaces by bacteria can occur in a matter of 2 weeks and reports state that there is a difference in the total bacterial load between peri-implant biofilms and peri-implant mucositis. Complex ecosystem made up of a variety of organisms that are typically dominated by gram-negative anaerobic bacteria. The peri-implant biofilm has recently been discovered to be a complex ecosystem made up of mixed, relatively changeable, and most often dominated by gram negative anaerobic bacteria as a result of enhanced sample processing techniques [18. Based on the evidence currently available, the following strains were taken into consideration for this study: Porphyromonas gingivalis, *Tannerella forsythia*, *Treponema denticolal*, *Aggregatibacter actinomycetemco mitans*, *Prevotella intermedia*, *Fusobacterium nucleatum*, *Campylobacter species*, and Bacteroides species. No metal or metal alloy is completely inert in vivo due to constant contact with tissues and body fluids, which in turn serves as a source for electrochemical interactions. Mechanical loading of the implant also results in ion loss by friction and electrochemical exchange, a process known as bio tribo-corrosion. It has also been proposed that long-term accumulation of biofilms and mechanical strain lead to implant surface degradation.

When compared to intervention-free sites, substantial quantities of dissolved titanium were seen in sub mucosal plaque around implants, showing a link between Ti dissolution and peri-implantitis. The oxide corrosion products were systemically distributed and were found in newly formed trabecular bone and peri-implant vasculature. The oxide particles tend to be cytotoxic, having an effect on immune cells, and it has been noted that the smaller the particle size, the more toxic the particle is. This particle also affects host immunity, activating macrophage and causing IL-1 to be produced as a result Osteolysis and osteo-clastogenesis are the results of a series of events. Other workers have observed peri implantitis to be present in situations where the microbial threat is removed or under control through frequent supportive measures, there is evidence which proposes that titanium oxide debris causes immune-modulatory changes which bring about degenerative changes in osseous and periodontal tissues, this is because of the fact that immune cells around the implant that is the polymorpho nuclear neutrophils, macrophages and monocytes recognize the implant as a foreign body, and release various signalling molecules such as reactive oxygen species, IL-8, TNFα, IL-6, IL-4, IL-10 which in turn affect the osteogenic capacity of the osteoblasts that adhere to that material surface (Vasconcelos *et al*. 2016), it has been proposed that surfaces roughness of the implant has a significant immuno modulatory effects and that the macrophages tended to polarize towards a classical M1 phenotype which upon activation are known to secrete high levels of pro-inflammatory cytokines. However it is interesting to note that TiO2 has a continuous photo-catalytic antimicrobial activity against pathogens this metal oxide alone or in combination with other metals like silver, copper or zinc is shown to have antimicrobial property and the same has been explored to a lesser extent, It can be assumed that titanium modifies the peri implant microbiome and has potential antibacterial activity because of the large number of interactions between TiO2 and common peri-implant pathogens we observed in the present study. The target was mostly enzymes involved in cellular nitrogen metabolism, which in turn caused alteration in protein synthesis and inhibited the ability of bacteria to cause virulence.

## Conclusion:

The occurrence of the inflammatory disease is occasionally unavoidable for any type of effective treatment plan. Molecular data that *Phyllanthus emblica* does indeed undergo degenerative changes and has the potential to modify the peri implant microflora by interfering with their metabolic processes and this could potentially increase the auto-immune response.

## Author contribution:

The first author (S Karthickraj) performed the analysis and interpretation and wrote the manuscript. Second author (R Subhashree) contributed to conception, data design, analysis, interpretation and critically revised the manuscript. Third author (N Thiyaneswaran) critically reviewed the manuscript. All the authors have discussed results and revised the manuscript.

## Figures and Tables

**Table 1:** Virulence level of various microbes in ascorbic acid

**Organism**	**Identifier**	**Protein interaction with amlaphyto derivative**	**Functional class**	**Virulency**	**Virulence prediction Score**
*Porphyromonas gingivalis*	Sod	superoxide dismutase	metabolism molecule	virulent	0.8488
*C. gracilis*	CAMGR0001_2267	superoxide dismutase	cellular process	virulent	0.8235
*Treponemadenticola*	TDE0238, trx	thioredoxin	cellular process	virulent	1.0729
*Treponema forsythia*	sodB	superoxide dismutase	cellular process	virulent	0.0679
*Enterococcus faecalis*	sgaB	phosphotransferase enzyme II	cellular process	virulent	1.0395
